# Effects of genotype and temperature on accumulation of plant secondary metabolites in Canadian and Australian wheat grown under controlled environments

**DOI:** 10.1038/s41598-017-09681-5

**Published:** 2017-08-22

**Authors:** Maryam Shamloo, Elizabeth A. Babawale, Agnelo Furtado, Robert J. Henry, Peter K. Eck, Peter J. H. Jones

**Affiliations:** 1 Richardson Centre for Functional Foods and Nutraceuticals, University of Manitoba, Winnipeg, MB R3T 2N2 Canada; 2 Queensland Alliance for Agriculture and Food Innovation, The University of Queensland, St Lucia QLD, 4072 Australia; 3 Department of Food Science, University of Manitoba, Winnipeg, MB R3T 2N2 Canada; 4 Department of Human Nutritional Sciences, W569 Duff Roblin Building, 190 Dysart Road, University of Manitoba, Winnipeg, MB R3T 2N2 Canada

## Abstract

Predictions of global increased temperature are for 1.8–4 °C by 2100. Increased temperature as an abiotic stress may exert a considerable influence on the levels of secondary metabolites in plants. These secondary metabolites may possibly exert biological activities beneficial in prevention or treatment of disorders linked to oxidative stress in human. Wheat secondary compounds in three Canadian and three Australian genotypes grown under controlled environments, in which the only changing parameter was temperature, were investigated. Kennedy and AC Navigator contained the highest amount of total phenolic acids among Australian and Canadian wheat genotypes, respectively. The total phenolic acids and total flavonoid contents of wheat genotypes increased following the increase of the growing temperature. In all the wheat genotypes, regardless of their growing temperatures, linoleic acid (C18:2n6) was measured as the main fatty acid. Significant increases in palmitic acid (C16:0) and oleic acid (C18:1n9) and significant decreases in linoleic acid (C18:2n6) and linolenic acid (C18:3n3) were observed at increased of growing temperature for all wheat genotypes. Growing temperature decreased campesterol content of wheat genotypes. Genotype and growing temperature significantly shifted the production of wheat secondary metabolites. This information might be used as a guide for breeding wheat varieties with higher antioxidant properties.

## Introduction

Wheat (*Triticum* spp.) is a major food cereal in the world grain market. The total global wheat production was around 711.2 million tons in 2013^1^. Some wheat genotypes have been reported to have high levels of secondary bioactive metabolites including phenolic acids (PAs), flavonoids, and phytosterols^2^. The phenolic group in polyphenols and flavonoids can accept an electron to form relatively stable phenoxyl radicals, thereby disrupting chain oxidation reactions in cellular components and therefore, limit the risk of various degenerative diseases associated with oxidative stress such as diabetes, chronic cardiovascular diseases and cancer^3–5^. Genotype and growing factors such as temperature and light affect the levels of phenolics and phytosterols in wheat grains as previously reported^6, 7^. However, most studies only evaluated the polyphenols and phytosterols of wheat grains grown in different geographical farm fields, while information on how genotypes and temperature impact the secondary metabolites levels of wheat grown under controlled conditions is limited.

As defined by the Intergovernmental Panel on Climate Change (IPCC, 2007), global warming relates to the increase in mean temperature that has been observed since the mid-twentieth century resulting from anthropogenic emissions of greenhouse gases into the atmosphere^8^. The IPCC’s fourth assessment predicted that the global temperature will increase by 1.8 to 4.0 °C by 2100. Increased temperature as an abiotic stress may exert a considerable influence on the levels of secondary metabolites in plants including wheat grains^9^. Therefore, considering the rapidly accelerating environmental changes on local and global scales, it is important to understand how secondary metabolites levels and composition change by increased temperature as such knowledge may have significant implications on nutritional values of wheat grains.

In order to adapt to temperature stress, plants use different protective mechanisms ranging from structural to biochemical^10^. One of the important biochemical defense mechanism is through enhanced production of secondary compounds. Phenolic acids and flavonoids are two important groups of plant secondary compounds, which are suggested to protect plants against abiotic stresses such as drought or increased temperature through their antioxidant properties to remove reactive oxygen species (ROS) before they oxidize cell walls and membranes^11, 12^. Elevated temperatures could increase the production of phenolics and flavonoids in plants, probably through the activation of their catalytic enzymes^13^. For instance, strawberry grown at increased temperature regimes had higher values of phenolic acid, flavonols, anthocyanins, and antioxidant capacities^14^. Jeong *et al*.^15^, reported a synergistic effect between high night-time temperatures and cultivation duration which produced lettuce rich in polyphenols compared to that at low temperature. Similarly, more soluble phenolics were measured in sugarcane (*Saccharum officinarum*) sprouts grown at 40/35 °C than those grown at 28/23 °C (day/night) temperatures^16^. However, to our knowledge, information concerning the influence of increased temperature on secondary compounds within cereal grains, including wheat, remains unknown. The purpose of the current study, therefore, was to investigate whether different wheat genotypes and/or growing temperatures could alter the accumulation of secondary metabolites in wheat grains differing in their genetic background.

## Results

### Yield

The durum wheat variety AC Navigator, which was developed for the Canadian prairies, when grown at 30 °C did not develop grains. All other varieties prevalent in Canada (AC Crystal and Carberry), or Australia (Kennedy, Fango60, and EGA Gregory) did develop grains at 20 °C, 25 °C and 30 °C.

Table 1, summarizes the grain yield in each temperature regime. Increased temperatures resulted in decreased wheat grain yields for all genotypes and for AC Navigator, there was no grain produced at 30 °C. Adverse impact of higher temperatures on grain yields are in line with previous studies^17–19^.

**Table 1:** Thousand kernel weight (g) of six wheat varieties grown in controlled temperatures.

Growing Temperature	Genotypes					
	AC Crystal	AC Navigator	Carberry	Kennedy	Fango60	EGA Gregory
20 °C	46.5^Ac^	43.35^Ab^	51.82^Bb^	44.93^Ac^	57.16^Cc^	46.0^Ab^
25 °C	38.89^Bb^	32.09^Aa^	33.33^Aa^	36.68^Ab^	48.34^Cb^	42.21^Bb^
30 °C	22.45^Aa^	na	29.32^Ba^	28.56^Ba^	35.67^Ca^	26.76^Aa^

^A,B,C,D^Different superscripts capital letters in the same row indicate significant difference (*P* < 0.05).

^a,b,c^Different superscripts small letters in the same column indicate significant difference (*P* < 0.05).

na = data not available, no grain produced.

### Phenolic Acids

Phenolic acids (PA), in each of the six wheat varieties grown at three temperatures are listed in Table 2. The mean value of total PAs, ranged from 389.54 ± 32.36 (Fango60, 20 °C) to 1007.61 ± 87.32 mg/kg dm (Kennedy, 30 °C). The highest content of total PAs, was observed for the genotype of Kennedy grown at 30 °C (1007.61 ± 87.32 mg/kg dm), followed by Kennedy grown at 25 °C (906.56 ± 46.17 mg/kg dm), and AC Navigator grown at 25 °C (829.41 ± 95.17 mg/kg dm). The lowest value was determined for Fango60 grown at 20 °C (389.54 ± 32.36 mg/kg dm). Based on the wheat genotypes, the mean value of total PAs was as follows: Kennedy > AC Navigator > AC Crystal > Carberry > EGA Gregory > Fango60.

**Table 2:** Free, bound and total phenolic acids content (microgram per gram of dry matter (dm)) in the whole grain of six wheat varieties grown in controlled environments.

Growing Environment	Phenolic acids	Genotypes					
		AC Crystal	AC Navigator	Carberry	Kennedy	Fango60	EGA Gregory
20 °C	Free	6.52 ± 0.02^Ea^	7.04 ± 0.02^Fa^	5.82 ± 0.03^Ca^	6.27 ± 0.01^Da^	5.02 ± 0.03^Aa^	5.48 ± 0.03^Ba^
	Bound	701.33 ± 36.63^Ca^	850.6 ± 50.9^Da^	546.23 ± 16.27^Ba^	674.33 ± 55.67^Ca^	384.51 ± 32.33^Aa^	493.87 ± 41.91^ABa^
	Total	707.85 ± 36.64^Ca^	857.65 ± 50.9^Da^	552.06 ± 16.25^Ba^	680.61 ± 55.66^Ca^	389.54 ± 32.36^Aa^	499.36 ± 41.88^ABa^
25 °C	Free	9.9 ± 0.01^Eb^	9.35 ± 0.02^Db^	9.19 ± 0.03^Cb^	10.99 ± 0.04^Fb^	8.04 ± 0.02^Ab^	8.45 ± 0.03^Bb^
	Bound	723.60 ± 84.31^Ca^	820.06 ± 95.1^CDa^	614.13 ± 62.6^BCab^	895.60 ± 46.15^CDb^	465.4 ± 39.3^ABab^	526.46 ± 58.36^ABa^
	Total	733.50 ± 84.3^Ca^	829.41 ± 95.17^CDa^	623.33 ± 62.58^BCab^	906.56 ± 46.17^CDb^	473.44 ± 39.31^ABab^	534.92 ± 58.38^ABa^
30 °C	Free	14.48 ± 0.04^Dc^	na	13.7 ± 0.04^Cc^	16.61 ± 0.03^Ec^	12.31 ± 0.01^Ac^	12.69 ± 0.01^Bc^
	Bound	743.62. ± 47.3^Ca^	na	698.27 ± 31.97^BCb^	991.0 ± 87.3^Db^	557.66 ± 37.9^Ab^	600.86 ± 55.02^ABa^
	Total	758.10 ± 47.32^Ca^	na	711.96 ± 32.01^BCb^	1007.61 ± 87.32^Db^	569.98 ± 37.98^Ab^	613.55 ± 55.01^ABa^

^A,B,C,D,E,F^Different superscripts capital letters in the same row indicate significant difference (*P* < 0.05).

^a,b,c^Different superscripts small letters in the same column in the same dependent variable indicate significant difference (*P* < 0.05).

na = data not available, no grain produced.

Irrespective of genotype, wheat grains grown at 30 °C produced higher amount of PAs followed by those grown at 25 °C, and 20 °C, respectively, except for the genotype of AC Navigator, in which, the total PAs of wheat grown at 20 °C was higher than 25 °C, and where no grain was produced by the plant at 30 °C. Increased growing temperature, resulted in a significant (*P* < 0.05) increase in free phenolics for all genotypes, as the free phenolics of all genotypes grown at 30 °C was twice to three times more than that for those grown at 20 °C. In addition, in all wheat samples grown, without considering the genotypes and the growing temperature, the contents of bound phenolics were significantly higher than free ones (*P* < 0.001).

**Figure 1:**
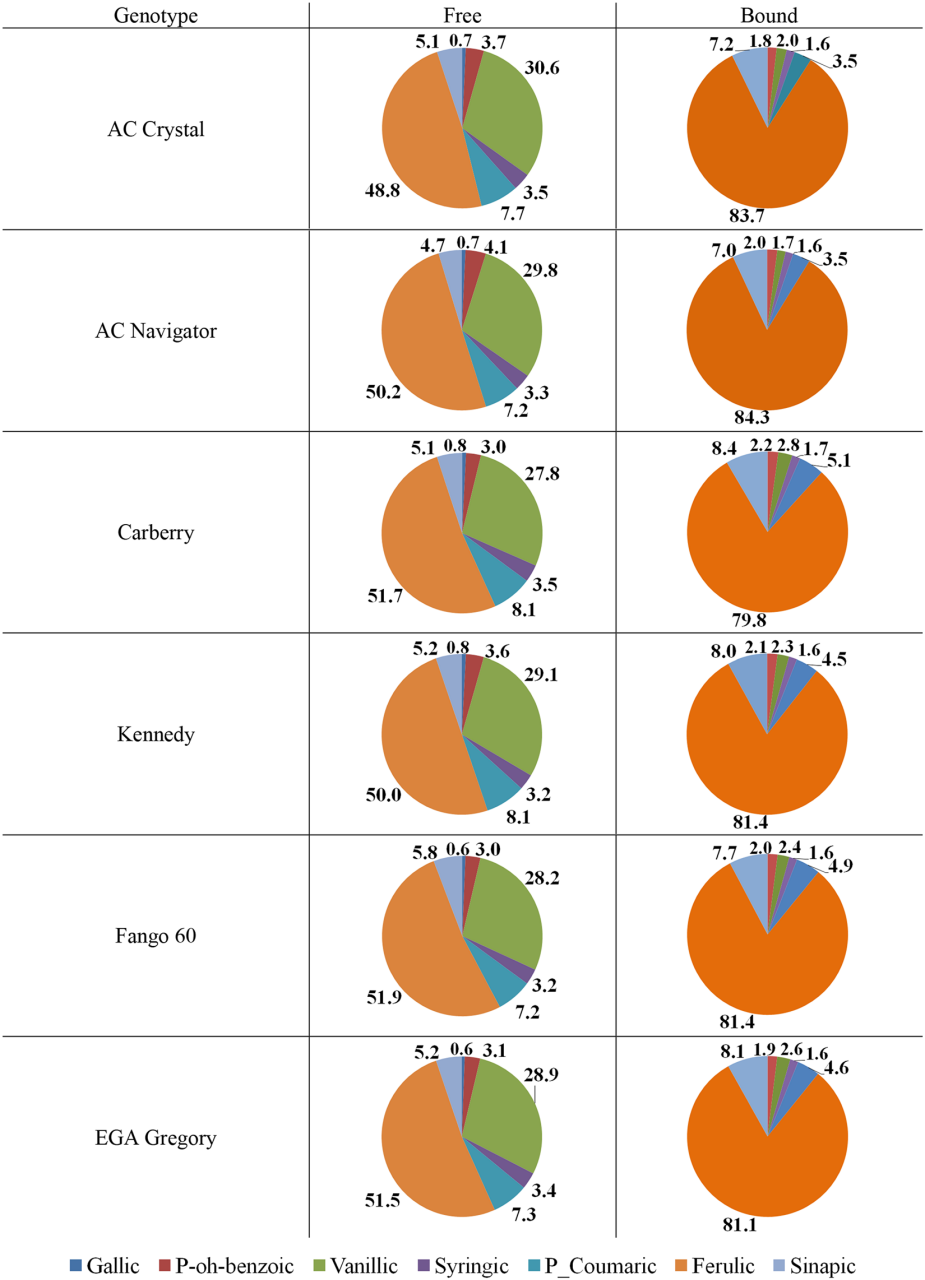
Relative distribution (%) of individual phenolic acids across free and bound fractions of six wheat varieties grown at 20 °C.

**Figure 2:**
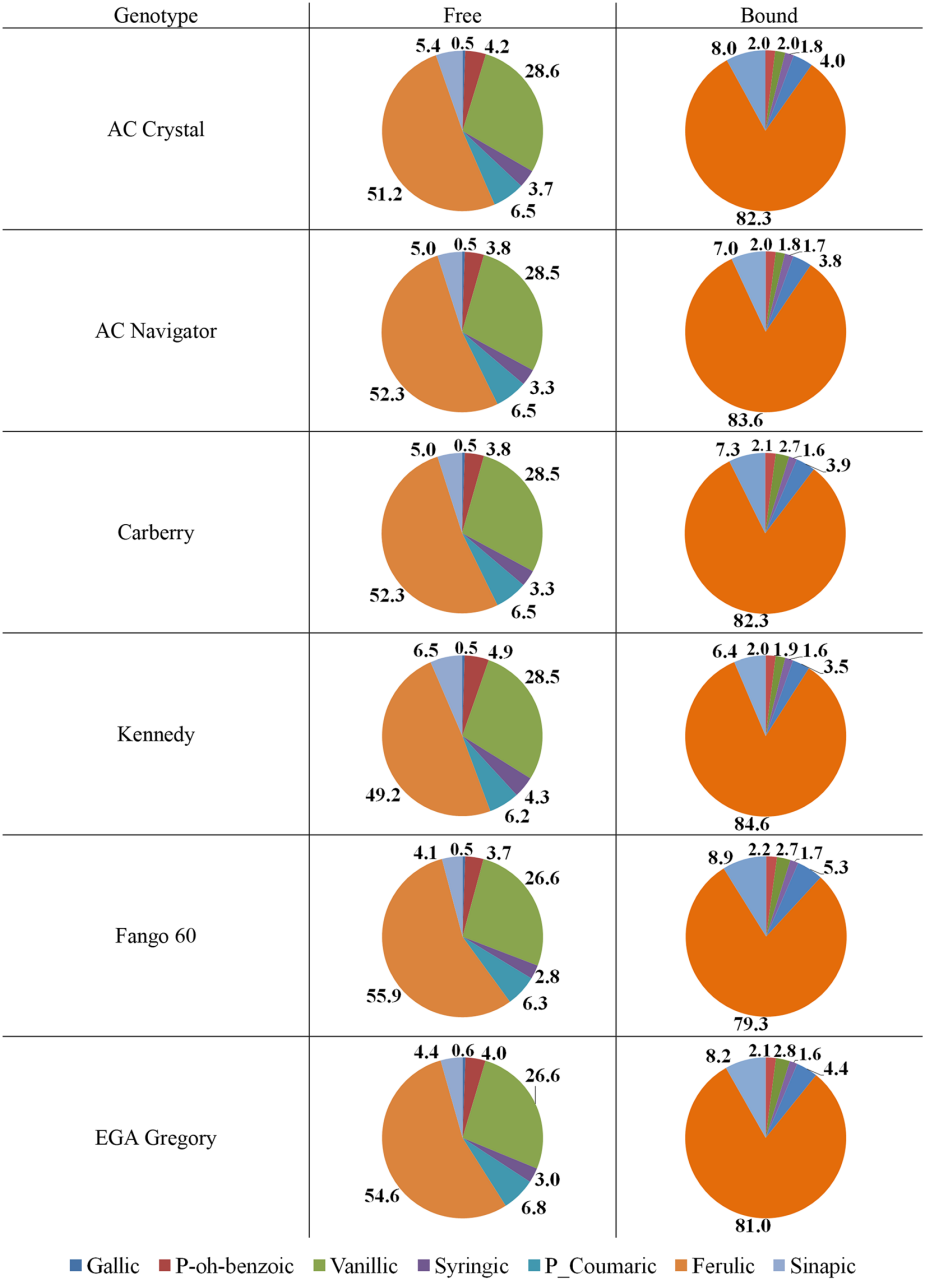
Relative distribution (%) of individual phenolic acids across free and bound fractions of six wheat varieties grown at 25 °C.

**Figure 3:**
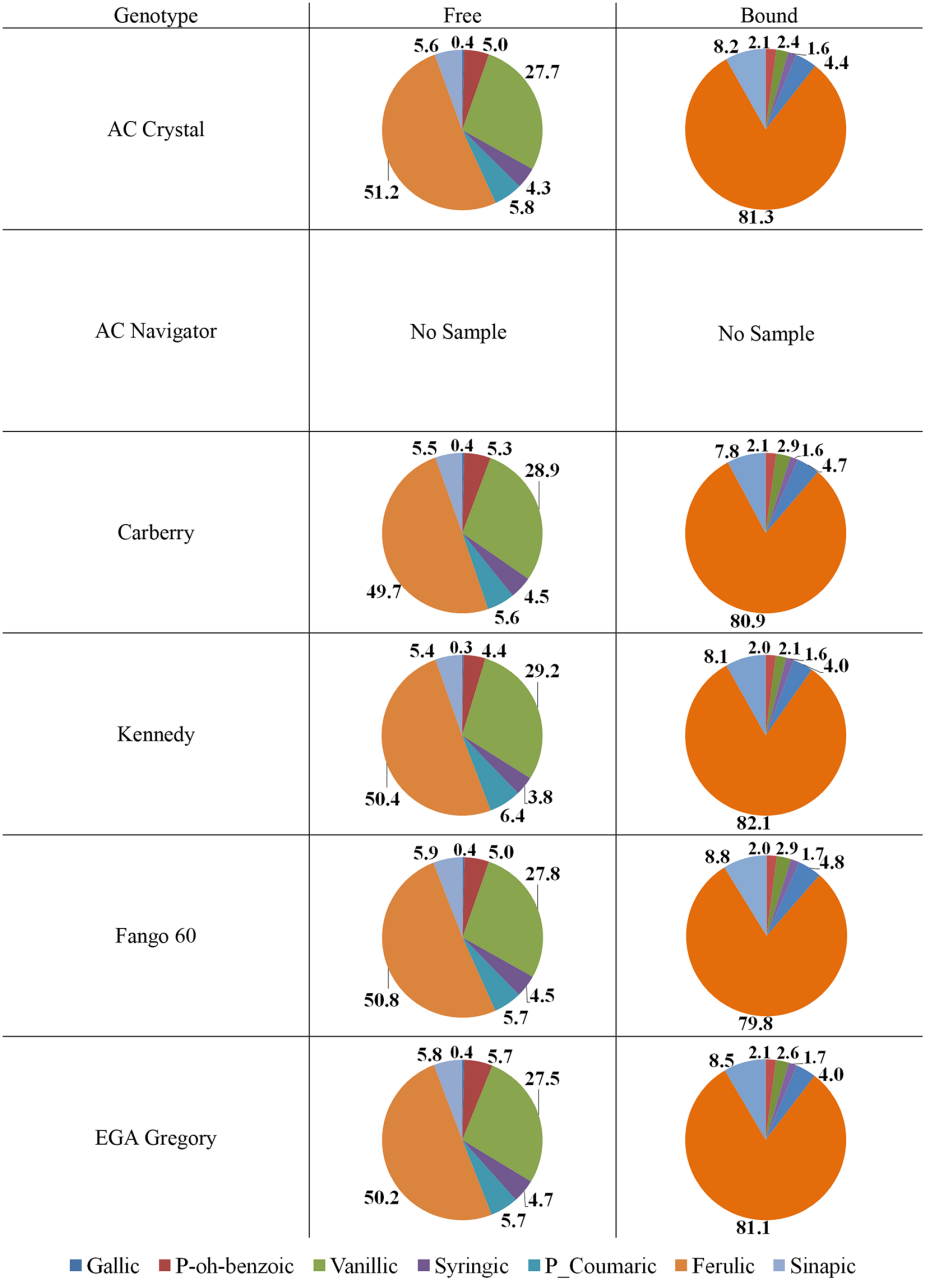
Relative distribution (%) of individual phenolic acids across free and bound fractions of six wheat varieties grown at 30 °C.

The relative distribution of individual identifiable PAs across free and bound phenolic acid fractions for different wheat genotypes grown at 20 °C, 25 °C, and 30 °C are shown in Figs 1, 2 and 3, respectively. In the bound PA fraction, ferulic acid contents were highest across all varieties, averaging 81.7%, followed by sinapic acid (7.8%), p-coumaric acid (4.3%), vanillic acid (2.4%), p-hydroxybenzoic acid (2.0%), and lastly by syringic acid (1.6%). The relative distribution of bound PA did not significantly differ between wheat varieties. For free PA, ferulic acid was most abundant, averaging 51.4%, followed by vanillic acid (28.4%), p-coumaric acid (6.7%), sinapic acid (5.3%), p-hydroxybenzoic acid (4.2%), syringic acid (3.7%) and gallic acid (0.5%). The relative distribution of free PA did not significantly differ between wheat varieties.

### Flavonoids

**Figure 4:**
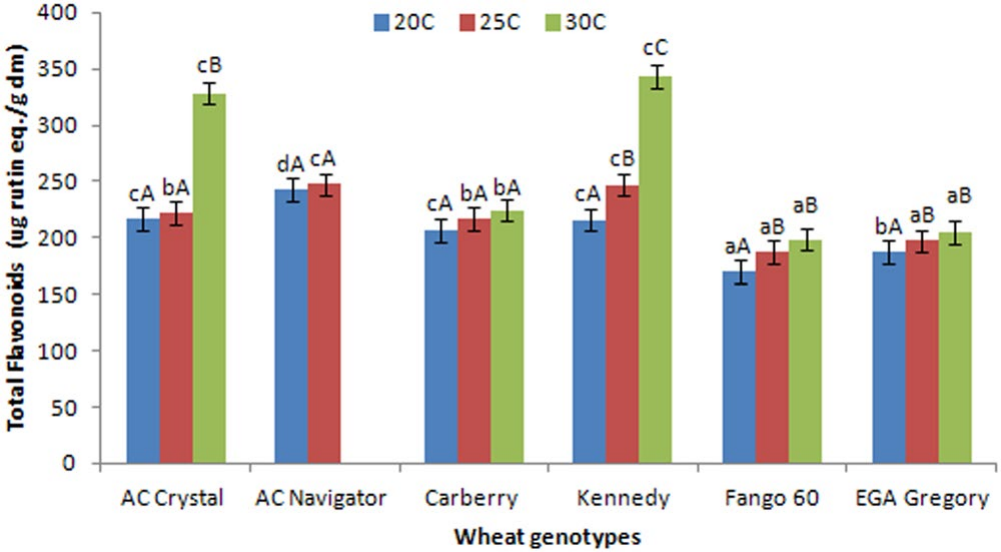
Total flavonoid contents (microgram rutin equivalent per gram of dry matter) of six wheat genotypes grown in controlled environments. ^a,b,c^Different small letter superscripts indicate significant differences (*P* < 0.05) between genotypes grown at the same temperature. ^A,B,C^Different capital letters superscripts indicate significant differences (*P* < 0.05) within the same genotype grown under different temperatures. (Data not available for AC Navigator grown at 30 °C).

Figure 4, shows the total flavonoid content in the grains of six wheat varieties grown in controlled environments. All genotypes showed significantly (*P* < 0.05) increased total flavonoids at increased growing temperatures. The lowest amounts of total flavonoid contents were observed in Fango60 and EGA Gregory grown at 20 °C, with 170.05 ± 2.24 and 187.59 ± 2.48 μg rutin equivalent/g dm, respectively. The highest amounts of total flavonoids were observed in Kennedy and AC Crystal grown at 30 °C, with 343.23 ± 3.03 and 328.6 ± 8.67 μg rutin equivalent/g dm, respectively.

### Fatty acids

Total fatty acid profiles of the six wheat genotypes grown under the different controlled environments are summarized in Table 3. All fatty acid concentrations were calculated as percentage values of total identified fatty acids measured. In all wheat genotypes, regardless of their growing temperatures, linoleic acid (C18:2n6) was measured as the main fatty acid ranged from 49.47% (EGA Gregory grown at 30 °C) to 57.73% (Carberry grown at 20 °C). Significant increases in palmitic acid (C16:0) and oleic acid (C18:1n9) and significant decreases in linoleic acid (C18:2n6) and linolenic acid (C18:3n3) were observed at increased growing temperatures for all wheat genotypes. Polyunsaturated fatty acids (PUFA) ranged from 50.4 to 59% of fatty acids profile and were two to three times greater than that of monounsaturated fatty acids (MUFA). The wheat varieties displayed different PUFA compositions regardless of growing conditions (Table 3 **)**. Generally, unsaturated fatty acid levels were three to four times higher than saturated fatty acid levels.

**Table 3:** Contents (%) of fatty acid profile of six wheat genotypes grown under controlled environments.

Growing Temperature	Fatty acids	Genotypes					
		AC Crystal	AC Navigator	Carberry	Kennedy	Fango60	EGA Gregory
20 °C	C16:0	20.26^a^	17.73^a^	18.35^a^	18.16^a^	19.03^a^	19.36^a^
	C18:0	0.92	1.04	1.31	1.2	0.95	1.11
	C18:1n9	16.74^a^	18.88^a^	16.96^a^	19.05^a^	16.76^a^	19.09^a^
	C18:2n6	55.74^a^	56.82^a^	57.73^a^	55.81^a^	57.55^a^	54.98^a^
	C18:3n3	4.42^a^	3.4^a^	3.56^a^	3.29^a^	3.99^a^	4.08^a^
	Others	1.91	21.12	2.1	2.49	1.7	1.37
	SFA	21.59	19.72	20.87	20.86	21.12	21.87
	MUFA	17.08	19.43	19.47	16.95	18.16	24.24
	PUFA	56.86^A^	57.48^A^	55.53^AB^	59.05^A^	57.19^AB^	51.75^B^
	UFA	78.41	80.28	79.13	79.14	78.88	78.13
	UFA/SFA	3.63	4.07	3.79	3.79	3.74	3.57
25 °C	C16:0	20.18^ab^	17.90^a^	19.49^ab^	19.07^ab^	21.00^ab^	19.56^ab^
	C18:0	0.91	1.27	0.98	1.03	1.44	1.36
	C18:1n9	16.01^a^	20.18^a^	16.79^a^	19.86^a^	16.62^a^	17.8^a^
	C18:2n6	57.4^a^	55.86^a^	57.33^a^	55.71^a^	56.03^a^	56.06^a^
	C18:3n3	3.81^ab^	2.85^ab^	3.07^ab^	2.97^ab^	3.29^ab^	3.51^ab^
	Others	1.7	1.94	2.34	1.37	1.61	1.7
	SFA	19.13	20.18	21.29	20.29	21.95	22.18
	MUFA	19.45	17.10	16.33	20.46	18.35	21.85
	PUFA	57.96^A^	58.70^A^	58.54^AB^	56.27^A^	56.21^AB^	53.10^B^
	UFA	80.87	79.82	78.71	79.71	78.05	77.82
	UFA/SFA	4.23	3.96	3.7	3.93	3.56	3.51
30 °C	C16:0	20.37^b^	na	20.94^b^	20.32^b^	20.46^b^	20.84^b^
	C18:0	1.17	na	1.10	1.15	1.3	1.34
	C18:1n9	17.99^b^	na	23.66^b^	23.76^b^	21.42^b^	23.97^b^
	C18:2n6	55.11^b^	na	49.51^b^	50.74^b^	52.06^b^	49.47^b^
	C18:3n3	3.42^b^	na	2.33^b^	2.59^b^	2.81^b^	2.67^b^
	Others	1.93	na	2.45	1.44	1.94	1.34
	SFA	20.02	na	19.52	23.07	22.47	22.6
	MUFA	17.46	na	20.58	16.95	24.13	24.45
	PUFA	58.89^A^	na	56.98^AB^	56.59^A^	50.50^AB^	50.46^B^
	UFA	79.98	na	78.71	76.93	77.53	77.4
	UFA/SFA	4.00	na	3.7	3.33	3.45	3.43

^a,b^Different small letters superscripts indicate significant differences in the same column in the same dependent variable (*P* < 0.05).

^A,B^Different capital letters superscripts in the same row indicate significant differences (*P* < 0.05). na = data not available.

### Phytosterol profiles

Table 4 shows the phytosterol compositions of 6 wheat genotypes grown under controlled environments. The total phytosterol content ranged from 604.5 μg/g dm (AC Crystal, 30 °C) to 1120.2 µg/g dm (AC Navigator, 25 °C). Sitosterol was the most abundant phytosterol in all wheat genotypes, regardless of their growing temperatures, accounting for 38–45% of the total sterols, followed by campesterol (14–18%), sitostanol (15–21%), campestanol (12–18%) and stigmasterol (1–3%). Growing temperature significantly decreased (*P* < 0.05) campesterol content of wheat genotypes growing at 30 °C compared to 25 °C, while other phytosterols did not show significant variation due to the growing environment. Overall, the wheat grown at 25 °C contained higher amounts of phytosterols compared to other temperatures. Among the wheat genotypes, the mean value of total phytosterols follows AC Navigator (1081.5 µg/g dm) > Kennedy (922.7 µg/g dm) > Fango60 (896.0 µg/g dm) > Carberry (769.4 µg/g dm) > AC Crystal (734.1 µg/g dm) > EGA Gregory (725.7 µg/g dm). The variation was much higher when it came to wheat genotypes for each growing environment, as wheat genotypes significantly (*P* < 0.05) altered all the individual phytosterols regardless of growing conditions as presented in Table 4.

**Table 4:** Phytosterol contents (microgram per gram of dry matter) of six wheat genotypes grown under controlled environments.

Growing Temperature	Plant sterols	Genotypes					
		AC Crystal	AC Navigator	Carberry	Kennedy	Fango60	EGA Gregory
20 °C	Sitosterol	358.3^B^	395.9^BC^	316.1^AB^	423.7^C^	400.2^BC^	287.7^A^
	Campesterol	148.1^abB^	188.7^abC^	127.1^abAB^	169.4^abBC^	164.4^abB^	119.6^abA^
	Sitostanol	127.3^AB^	195.9^D^	128.9^AB^	158.4^C^	145.4^BC^	102.2^A^
	Campestanol	106.2^ABC^	188.8^D^	99.3^AB^	124.2^C^	118.05^BC^	87.5^A^
	Stigmasterol	12.27^AB^	22.9^D^	11.4^A^	15.0^C^	14.0^BC^	15.1^C^
	Others	37.6^B^	50.5^C^	30.8^A^	38.1^B^	38.3^B^	27.2^A^
	Total	790.1^BC^	1042.9^E^	713.8^AB^	929.1^DE^	880.5^CD^	639.6^A^
25 °C	Sitosterol	352.0^B^	428.7^D^	346.1^B^	449.7^D^	397.8^C^	299.4^A^
	Campesterol	140.2^aA^	192.0^aC^	136.7^aA^	182.7^aBC^	167.5^aB^	121.8^aA^
	Sitostanol	151.2^AB^	222.7^D^	162.6^BC^	209.6^D^	181.8^C^	131.4^A^
	Campestanol	110.2^A^	197.5^D^	114.8^AB^	150.0^C^	130.9^B^	103.0^A^
	Stigmasterol	12.4^A^	23.7^C^	11.5^A^	15.6^B^	12.9^AB^	13.3^AB^
	Others	41.6^AB^	55.4^C^	36.9^AB^	54.5^C^	47.8^BC^	30.9^A^
	Total	807.9^B^	1120.2^D^	809.0^B^	1062.2^D^	938.9^C^	700.0^A^
30 °C	Sitosterol	259.1^A^	na	360.2^BC^	349.3^B^	386.4^C^	370.0^BC^
	Campesterol	90.5^bA^	na	110.8^bB^	110.2^bB^	133.9^bC^	126.7^bC^
	Sitostanol	126.3^A^	na	156.3^B^	169.5^B^	175.0^B^	168.2^B^
	Campestanol	87.3^A^	na	97.8^AB^	100.6^BC^	109.7^C^	106.1^BC^
	Stigmasterol	11.1^A^	na	19.6^C^	15.2^B^	15.6^B^	19.4^C^
	Others	29.9^A^	na	41.0^B^	31.8^A^	48.0^B^	47.0^B^
	Total	604.5^A^	na	785.5^B^	776.9^B^	868.8^C^	837.6^BC^

^a,b^Values with different superscripts in  the same column in the same dependent variable are significantly different (*p* ≤ 0.05). ^A,B,C,D,E^Values with different superscripts in the same row are significantly different (*p* ≤ 0.05). Abbreviations; na = data not available.

### Effects of genotype and temperature on wheat phenolic acids

As presented in Table 5 genotype, environment and their interactions significantly (*P* < 0.01) influenced phenolic acid levels including free, bound and total phenolic acids.

**Table 5:** Analysis of variance: Influence of genotype, environment and genotype environment interaction, on each compound class and each individual metabolite of six wheat genotypes grown under controlled environments.

Classes/metabolites	ANOVA significance (Mean square values)		
	Genotype (*df* 5)	Environment (*df* 2)	G × E (*df* 10)
**Phenolic acids**			
Free phenolics	36.05*	142.9*	36.58*
Bound phenolics	176272*	30419*	159503*
Total phenolics	179838*	30483*	164322*
**Free phenolics**			
Gallic acid	0.0000003*	0.0000002**	0.0000004*
P-oh-benzoic	0.0000514*	0.0004060*	0.0000631*
Vanillic	0.0019348*	0.0066585*	0.0019014*
Syringic	0.0000425*	0.0002598*	0.0000448*
P-coumaric	0.0000925*	0.0001454*	0.0000808*
Ferulic	0.0053631*	0.0231092*	0.0058324*
Sinapic	0.0000922*	0.0003282*	0.0000000*
**Bound phenolics**			
P-oh-benzoic	0.0000167*	0.0000031*	0.0000166*
Vanillic	0.0000215*	0.0000037*	0.0000148*
Syringic	0.0000117*	0.0000032*	0.0000107*
P-coumaric	0.0000536*	0.0000029*	0.0000580*
Ferulic	0.031964*	0.006259*	0.027888*
Sinapic	0.0002175*	0.0000159**	0.0002247*
**Flavonoids**			
Total flavonoids content	15138*	815*	17127*
**Fatty acids**			
C16:0	94.349*	26.267*	62.917*
C18:0	0.20440*	ns	0.18133*
C18:1n9	73.53*	1.97**	100.637*
C18:2n6	450.44*	1110.41*	443.98*
C18:3n3	3.3321*	10.1768*	0.9317*
Others	0.58909*	0.54828*	0.69966*
SFA	106.797*	29.554*	72.615*
MUFA	76.350*	1.992**	104.922*
PUFA	468.21*	1152.94*	462.44*
UFA	935.5*	1267.9*	1049.1*
UFA/SFA	1.3828*	5.4187*	2.5248*
PUFA/MUFA	2.1351	8.7418*	0.8051*
**Plant sterols**			
Sitosterol	22446*	43208*	30529*
Campesterol	2109.1*	21312.0*	4786.1*
Sitostanol	3139.1*	9520.0*	8696.3*
Campestanol	1538.2*	12442.1*	5626.4*
Stigmasterol	19.263*	15.476*	124.931*
Other sterols	141.31*	618.75*	645.14*
Total plant sterols	74095*	325481*	198126*

An asterisk (*) indicates significant at *P* < 0.001; Two asterisks (**) indicate significant at *P* < 0.01; ns indicates not significant at *P* < 0.05. A bold number is the factor or interaction most influencing the variable (highest mean square for each variable); *df*: degrees of freedom. G × E: Genotypes, environment interactions.

### Effects of genotype and temperature on wheat flavonoids

Total flavonoids influenced significantly by genotype, environment and genotype-by-environment interaction. Genotype-by-environment interaction effect was larger than genotype or environment effect individually (Table 5).

### Effects of genotype and temperature on wheat fatty acids

Genotype variations had higher influence on C16:0, C18:0, and SFA concentrations, where C18:2n6, C18:3n3, PUFA, UFA, UFA/SFA, PUFA/MUFA levels were mostly influenced by environmental changes. Genotype-by-environment interactions had higher influence only on C18:1n9 and MUFA as indicated in Table 5. In general, the greater effect of environment compared to wheat genotypes was observed for fatty acid profiles.

### Effects of genotype and temperature on wheat phytosterols

Environment contributed to a higher extent on the total plant sterol level and on all individual sterols except for the stigmasterol, which was mostly influenced by genotype-by-environment interactions (Table 5).

## Discussion

This research provides new information on how genotype and growing temperature impact the production of wheat secondary metabolites, which can be used to produce the wheat products that contain higher amount of targeted bioactives.

Increased growth temperature from 18/12 °C to 30/22 °C (day/night) for strawberry, yielded fruit with the most phenolic contents^14^. Similarly, in the present study, the total phenolic acids of wheat genotypes increased following the increase of the growing temperature. The increased levels of total phenolic acids could be related to plant’s defense mechanism against temperature stress as also reported in other studies^10, 20^ and could be a response to the generation of ROS. Therefore, the wheat grains produced at higher temperatures possess higher antioxidant properties which is a positive nutritional enhancement. These possible health benefits of phenolic acids depend on their absorption and metabolism, which in turn are determined by their structure including their conjugation with other phenolics, degree of glycosylation/acylation, molecular size and solubility. For example, it was reported that phenolic acids, when ingested in the free form, are rapidly absorbed by the small intestine and therefore may have health benefits of protection against cardiovascular disease and certain types of cancer due to their antioxidant properties which are the lowering of the levels of free radicals present in the body^5, 21^. However, bound phenolic acids are naturally esterified in plant products and esterification impairs their absorption because intestinal mucosa, liver and plasma do not possess esterases, and therefore hydrolysis can be performed only by microflora present in the colon^5^. As these compounds reach the colon, they will be degraded by the colon microflora and may exert antioxidant activities, as several studies have linked microbial metabolism of bound phenolic acids to colon cancer prevention^5, 22, 23^.

The individual phenolic acids identified in the present study were similar to those observed in other research^24, 25^, including levels of five main phenolic acids: ferulic acid, vanillic acid, *p*-coumaric acid, sinapic acid, and syringic acid. As reported in a previous study^24^, the results of this work also suggest that bound phenolic acids in wheat, which included the major proportion of the total phenolic acids, were strongly affected by the genotype variation and less influenced by the environment. However, presently the environmental effect was larger than genotypic differences for free phenolic acids^24^. Other investigators observed different trends for environmental changes compared to genotypic variation; for example, the results of Mpofu *et al*., showed that environmental effects on the content of phenolic compounds were considerably larger than genotypic effects^26^. Although these investigators used different wheat genotypes in their study, the main possible reason could have been that these investigators collected their samples from different fields across Western Canada, where all the environmental factors including soil pH, temperature and rainfall were different. In contrast presently we used controlled environments in which the only changing parameter was temperature. The profile and levels of phenolic acids determined in the wheat genotypes in the present study were similar to those observed in previous work^7, 27–30^, confirming that biosynthesis of phenolic compounds in plants including wheat grains is under genetic control and is strongly influenced by biotic and abiotic factors such as temperature.

Total flavonoid contents of wheat measured in the present study were in agreement with those reported in previous work^31, 32^. Within the Canadian wheat genotypes AC Crystal contained higher amounts of flavonoids as expected: red spring wheat and colored wheat varieties have been reported to contain higher flavonoid content previously^31^. The increased growing temperature resulting in higher amount of total flavonoids can be explained as the effect of temperature forcing the plant to produce extra flavonoids as a defense strategy against the environmental changes. Enhanced levels of flavonoids suggest that the grains grown at higher temperatures possess not only higher total phenolic acids but they also have higher amounts of flavonoids which again increases the overall antioxidant properties of such grains. Such information could be used as useful strategies to produce wheat products with higher nutritional value considering the genotype and the temperatures during the growing season in different geographical environments such as Australia and Canada.

The main fatty acids identified in the wheat in the present study were similar to those reported previously^33–35^. Linoleic acid (C18:2n6) and linolenic acid (C18:3n3), two essential fatty acids, were both decreased with increased growing temperature for all wheat genotypes. Also, palmitic acid (C16:0), a saturated fatty acid, increased at higher temperatures. Increased levels of palmitic acid in the diet has been linked to elevated LDL level and thereby increased cardiovascular diseases^36^. Therefore, unlike the phenolic acids and flavonoids contents, the fatty acid profile of wheat grains produced under higher temperatures was negatively affected by temperature.

Although the growing environment, genotype, and genotype-by-environment interactions all significantly affected the SFA and UFA compositions of wheat, the genotypes variation had a higher influence on saturated fatty acids, while the unsaturated fatty acids were more affected by the environment changes. These findings are in agreement with results of Bleggia *et al*.^37^ who also observed a large effect of genotype-by-environment interactions on fatty acid profiles of wheat cultivars harvested across three cultivation years and two cultivation systems (conventional and organic), however, these investigators observed a non-significant effect for the genotype variation on SFA and UFA compositions. This finding could be related to differences in genotypes or growing conditions.

The most abundant phytosterol in all wheat genotypes was sitosterol which is in line with previous findings^38, 39^. The total phytosterol contents were also in the range as previously found^6, 39^. The genotype and environment and their interaction resulted in significant differences in the proportions of the individual plant sterols and total plant sterols which confirmed the previous report^6^. Environment effects were greater than the genotype variation for all individual plant sterols, except the stigmasterol which was highly influenced by genotype -by-environment interaction.

Overall, the effect of global warming on wheat secondary bioactive metabolites appears to be genotype-specific as well as dependent on the category of metabolite. However, some trends seem to be related to the type of environmental stressor as well. For example, in the present study the increase of phenolic compounds, including phenolic acids and flavonoids, agrees with data in the literature indicating that they are usually positively enhanced by an elevated temperature.

In conclusion, the profiles and contents of the secondary metabolites presently studied in wheat grains were significantly influenced by the genotype, growth environment and genotype by environment interactions. The comprehensive data set produced in this study constitutes a valuable basis to further our understanding of the variations of wheat bioactives grown under different temperatures, and enables the selection of particular wheat grains to be used as a nutritious food source depending on growth environment and genotype. For example, of all six genotypes used in the present study, Kennedy and AC Navigator contained higher amounts of phenolic acids and phytosterols within the Australian and Canadian wheat genotypes, respectively. Knowledge of such genotypic differences in phenolic acids and phytosterols can be used for breeding wheat varieties with higher antioxidant properties. Another example is that based on the present study, the Canadian genotype of AC Navigator will not yield at the increased temperature above 25 °C, which can be a useful information for wheat breeders.

The present study, however, has some limitations. The only variable parameter in the present work was the temperature and it can be expected that other environmental factors such as water deprivation, elevated CO2 and UV light can induce a different physiological response in plants. Therefore, it is difficult to predict the outcome of present and future climatic changes based on the evaluation of only one or two parameters at a time. Thus our present results are lacking information regarding the combined effect of these abiotic factors on wheat secondary metabolites. Future studies should therefore focus on simultaneously testing the effects of multiple environmental factors to gain a more realistic perspective of how global climatic changes may impact the production of secondary bioactive metabolites of wheat grains.

## Methods

### Experimental design

Three separate growth chambers (GR192) were used to control all environmental factors and provide three temperature regimes, at the Richardson Centre for Functional Foods and Nutraceuticals, University of Manitoba, Winnipeg, Canada, 2015, where the temperature was altered and other environmental factors including photoperiod, carbon-dioxide levels, humidity and wind velocity were fixed. The chambers shared a common, re-circulating nutrient solution developed for wheat. Photosynthetic photon flux (PPF) provided with cool-white, VII0 fluorescent lamps. The photoperiod was set to be 20-h. Temperature was maintained at 20 ± 0.2 °C, 25 ± 0.2 °C and 30 ± 0.2 °C in growth chambers 1, 2, and 3, respectively. Chamber CO_2_ concentration was controlled by mixing pure CO_2_ with outside air. Air flow into each plant growth chamber was maintained at 30 L min^−1^ to provide a rapid air turnover rate (once per minute).

The experiment was a completely randomized block design (using 6 wheat genotypes) within three temperature treatments and three replications (individual plants). Each variety was grown as three replicates and each replicate consisted of a minimum of five potted plants, i.e., a total of 15 plants per variety (as described in supplementary information). The seeds of each genotype were harvested at maturity, manually cleaned and air-dried until a moisture content of 10% ± 0.5 was reached. The dried samples from each replicate were individually vacuum-packed in moisture proof packaging and stored at −20 °C in the dark until analysis.

### Wheat genotypes

Six wheat genotypes (*Triticum spp*) were used in this study as follows:

#### Three Canadian wheat genotypes

1. AC Crystal, red spring wheat (*Triticum aestivum L*.), 2. AC Navigator durum wheat (*Triticum turgidum L. var. durum*), 3. Carberry, a hard red spring wheat (*Triticum aestivum L*.), were kindly donated from Cereal Research Centre, Agriculture and Agri-Food Canada, Winnipeg, MB, Canada.

#### Three Australian wheat genotypes

1. Kennedy, quick maturing spring wheat (*Triticum aestivum L*.), 2. Fango60, drought tolerant wheat (*Triticum aestivum L*.), 3. EGA Gregory, hard spring wheat (*Triticum aestivum L*.), were kindly donated from Queensland Alliance for Agriculture and Food Innovation, The University of Queensland, St Lucia, QLD, Australia.

### Sample preparation

The whole wheat samples were milled using an ultra centrifugal mill (Model ZM 200, Retsch, Haan, Germany) and passed through a 0.5mm sieve screen using rpm of 14,000. The fine flour from each sample was individually vacuum-packed in moisture proof packaging and stored at −20 °C in the dark until analysis. Four categories of bioactives, including phenolic acids, flavonoids, fatty acids and plant sterols, extracted separately, and analyzed from these growth chamber grown wheat grains.

### Free and bound phenolic acid extraction

The extraction was performed using liquid-liquid extraction and alkaline hydrolysis steps^40^ with slight modification. Briefly, Wheat flour (0.6 g) was extracted twice with Ethyl acetate at a ratio of 1:20 (w/v). Each time, the mixture was kept on a mechanical shaker (Thermo/Lab-Line/Barnstead MAX Q 5000, Artisian Scientific, Champaign, IL, USA) for 1 h at room temperature. After centrifuging (Model Sorvall Legend RT, Thermo Electron Corporation, Osterode, Germany) at 3750 *g* for 10 min, the supernatants obtained from each time were combined and concentrated to dryness by using an analytical nitrogen rotary evaporator (Model N-EVAP 112, Organomation Assocuates, Inc, Berlin, MA, USA) at 30 °C. The dried extract was re-suspended in 1.2 mL of 50% Dimethyl sulfoxide (DMSO)-Ethanol as crude extracts and kept in a sealed amber vial container at 4 °C. This extract was referred to as free fraction. The dried residue obtained from crude extraction was hydrolyzed with 18 mL of 4 M NaOH on a shaker (Thermo/Lab-Line/Barnstead MAX Q 5000, Artisian Scientific, Champaign, IL, USA) under nitrogen gas for 4 h. After digestion, the solution was adjusted to a pH 1.5–2.0 with 6 M ice cold HCl and then extracted with 12 mL of ethyl acetate three times. After centrifuging (Model Sorvall Legend RT, Thermo Electron Corporation, Osterode, Germany) at 3750 *g* for 10 min, the combined ethyl acetate fractions were evaporated to dryness and reconstituted in 1.2 mL of 50% Dimethyl sulfoxide (DMSO)-Ethanol and kept in a sealed amber vial container at 4 °C. This extract obtained from residues was referred as bound fraction. Both fractions were directly subjected to HPLC analysis. Prior to HPLC analysis, they were filtered through a 0.45 μm syringe filter.

### HPLC-PDA analysis

The HPLC (Waters 2695, Milford, MA, USA) equipped with a photodiode array detector (PDA) (Waters 996), and auto-sampler (717 plus, Waters, Milford, MA, USA) used to analyze phenolic acids. A 250 × 4.6 mm, 5 µm RP 18 column (Shim-pack HRC-ODS, SHIMADZU Corp., Tokyo, Japan) was used for separation. Each sample (20 µl) was injected via an auto-sampler, and eluted through the column with a gradient mobile phase consisting of A (0.1% acetic acid in water) and B (0.1% acetic acid in methanol) with a flow rate of 0.5 ml/min.

A 75 min linear gradient was programmed as follows: 0–11 min, 9–14% B; 11–14 min, 14–15% B; 14–17 min, 15% B;17–24 min, 15–16.5% B; 24–28 min, 16.5–19% B; 28–30 min, 19–25% B; 30–36 min, 25–26% B; 36–38 min, 26–28% B; 38–41 min, 28–35% B; 41–46 min, 35–40% B; 46–48 min, 40–48% B; 48–53 min, 48–53% B; 53–70 min, 53–70% B; 70–72 min, 70–9% B; 72–75 min; 9% B. The peaks of phenolic acids were detected at a wavelength of 280 nm. The quantification of phenolic acid content were calculated using external calibration curves of gallic acid (0.001 to 0.01 mg/ml, equation: y = 0.0021x + 1.0216), protocatechiuic acid (0.001 to 0.01 mg/ml, equation: y = 0.0036x + 0.3842), p-hydroxybenzoic acid (0.00099 to 0.0099 mg/ml, equation: y = 0.0022x + 0.2571), vanillic (0.001 to 0.01 mg/ml, equation: y = 0.0017x + 0.6503), caffeic (0.001 to 0.01 mg/ml, equation: y = 0.0022x + 0.2152), syringic (0.001 to 0.01 mg/ml, equation: y = 0.001x + 0.2548), p-coumaric (0.001 to 0.01 mg/ml, equation: y = 0.0007x + 0.3395), ferulic (0.001 to 0.01 mg/ml, equation: y = 0.0011x + 0.2972), sinapic (0.001 to 0.01 mg/ml, equation: y = 0.0025x + 0.2613), isoferulic (0.001 to 0.01 mg/ml, equation: y = 0.0009 x + 0.2747), o-coumaric (0.001 to 0.01 mg/ml, equation: y = 0.0006 + 0.1171). The HPLC retention times of 11 different phenolic acids standards and a representative chromatogram of a wheat extract are presented in supplementary information.

### Total flavonoid content

Flavonoid contents of wheat fractions were assayed using the aluminum chloride colorimetric method of Chang *et al*.^41^. The appropriate dilution of extracts (0.5 ml) were mixed with1.5 ml of 95% ethanol, followed by 0.1 ml of 10% aluminum chloride, 0.1 ml of 1 M potassium acetate and 2.8 ml of distilled water. After incubation at room temperature for 30 min, the absorbance of the reaction mixture was measured at 415 nm with a UV/Visible spectrophotometer (Model DU 800, Beckman Coulter, Inc., CA, USA). The flavonoid content was calculated using a standard calibration of rutin solution and expressed as micrograms of rutin equivalent (RE) per gram of sample.

### Fatty acid profile analysis

Fatty acids were extracted as described by Tsen *et al*.^42^, adopted from the classic Folch method^43^,using chloroform-methanol (2:1, volume to volume (v/v)) containing 0.01% butylated hydroxytoluene (Sigma-Aldrich, Oakville, Ontario, Canada), followed by methylation with methanolic HCl. Fatty acid methyl esters were then analyzed using an Agilent 6890 N (Agilent Technologies, Mississauga, ON, Canada) gas chromatograph equipped with a flame ionization detector. During the extraction and methylation, heptadecanoic acid (C17:0) was used as an internal standard (Sigma-Aldrich, Oakville, Ontario, Canada). Known fatty acid standards (Sigma-Aldrich, Oakville, Ontario, Canada) were used to identify the individual fatty acids in wheat samples. The level of each fatty acid was then calculated according to the corresponding peak area relative to the total area of total interested fatty acids, and considered as a percentage of the total fatty acids.

### Phytosterol extraction and derivatization

The procedure used for phytosterol extraction included acid and alkaline hydrolyses and was based on the method of Piironen *et al*.^44^. The internal standard dihydrocholesterol (DHC, 40 μg) was first added into a 0.5 gram cereal sample. The sample was then subjected to acid hydrolysis with hydrochloric acid (HCl) to liberate sterols from their glycosidic conjugates. After acid hydrolysis and the extraction of lipids, alkaline hydrolysis with potassium hydroxide (KOH) saponified the lipids and hydrolysed the esterified sterols into free sterols. The unsaponifiable lipids (containing free sterols) were extracted into cyclohexane and purified by solid-phase extraction (SPE) using silica cartridges (Strata SI-1, 500 mg, Phenomenex, Torrance, CA, USA). Prior to the gas chromatographic analysis, phytosterols were derivatised to trimethylsilyl (TMS) ethers using N,O-bis (trimethylsilyl) trifluoroacetamide (BSTFA, Fisher Scientific, Grand Island, NY, USA) and trimethylchlorosilane (TMCS, Sigma Aldrich, Oakville, ON, Canada) in a ratio of 99:1 (v/v) as the reagents in anhydrous pyridine (Sigma Aldrich, Oakville, ON, Canada). Each sample was analyzed in duplicate.

### Phytosterol gas chromatographic analysis

Phytosterols were analyzed using an Agilent 6890 N gas chromatograph (Agilent Technologies, Mississauga, ON, Canada) with flame ionization detection (FID) and an on-column injector. The GC was equipped with a SAC-5 silica capillary column (30 m × 0.25 mm × 0.25 μm, Supelco Inc., Bellefonte, PA, USA). Peak identification was accomplished by comparing the retention times with those of a standard mixture of pure sterols. Quantification of phytosterols was performed using dihydrocholesterol as the internal standard.

### Statistical analysis

All data were reported as means ± SD of triplicate independent experiments. The main effects of genotype and environment and their interaction were investigated by one way or general linear model ANOVA with Minitab 14 Statistical software (Minitab Inc., State college, PA, USA). Significant differences were considered when (*P* < 0.05) unless stated otherwise.

### Data availability

All data generated or analysed during this study are included in this published article (and its Supplementary Information files).

## Electronic supplementary material

Supplementary Information
