# Supplementary material for: Effects of genotype and temperature on accumulation of plant secondary metabolites in Canadian and Australian wheat grown under controlled environments
Source: Sci Rep. 2017 Aug 22;7:9133. doi: 10.1038/s41598-017-09681-5 (PMC5567368; doi:10.1038/s41598-017-09681-5)
Supplement: Supplementary file 1 — Supplementary Information [file 41598_2017_9681_MOESM1_ESM.pdf]

Title Page:

Type of Article: Research Article

Title: Effects of genotype and temperature on accumulation of plant secondary metabolites in Canadian and Australian wheat grown under controlled environments

Authors: Maryam Shamloo[1,3], Elizabeth A Babawale[1], Agnelo Furtado[2], Robert J Henry[2], Peter K Eck[4] and Peter JH Jones[1,3]*

[1]*Richardson Centre for Functional Foods and Nutraceuticals,* University of Manitoba, Winnipeg, MB R3T 2N2, Canada
[2]Queensland Alliance for Agriculture and Food Innovation, The University of Queensland, St Lucia QLD 4072, Australia
[3]Department of Food Science, University of Manitoba, Winnipeg, MB R3T 2N2, Canada
[4]Department of Human Nutritional Sciences, W569 Duff Roblin Building, 190 Dysart Road, University of Manitoba, Winnipeg, MB, R3T 2N2, Canada

*__Corresponding author__: Peter JH Jones, [1]*Richardson Centre for Functional Foods and Nutraceuticals,* University of Manitoba, Winnipeg, MB R3T 6C5, Canada
Tel: +1(204) 474 8883, Email: Peter.Jones@umanitoba.ca

Title: Effects of genotype and temperature on accumulation of plant secondary metabolites in Canadian and Australian wheat grown under controlled environments

Growth Chambers (Temperatures: 20°C, 25°C, 30°C)

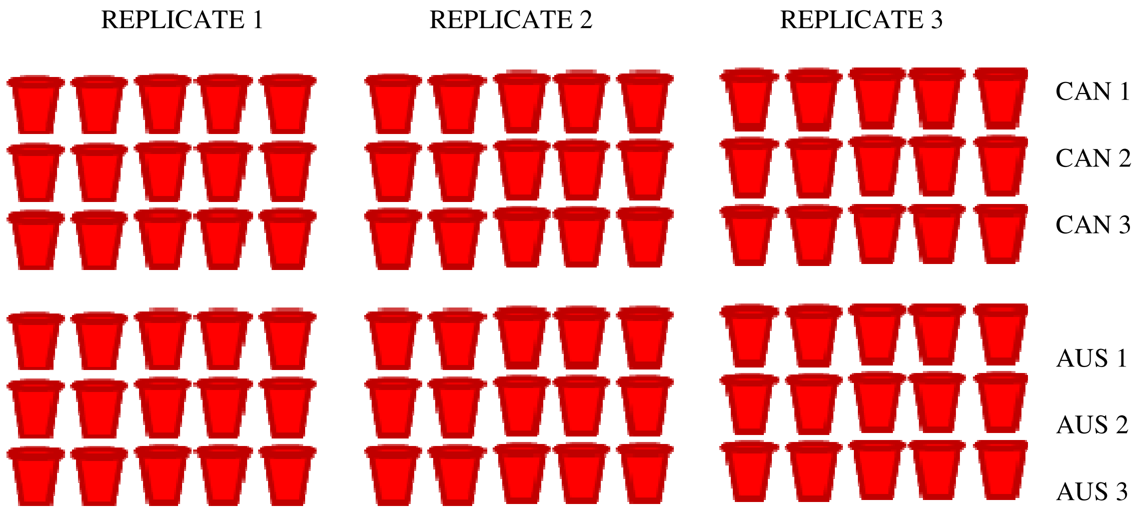

Supplemental figure 1: Pictorial representation for the layout of the temperature regimes experiment in growth chambers.

NOTE: Six varieties of 3 Australian (AUS) and 3 Canadian (CAN) within each replicate were arranged in a random fashion in accordance with the statistical procedures for the design of experiment as per Randomized Block Design (RBD).

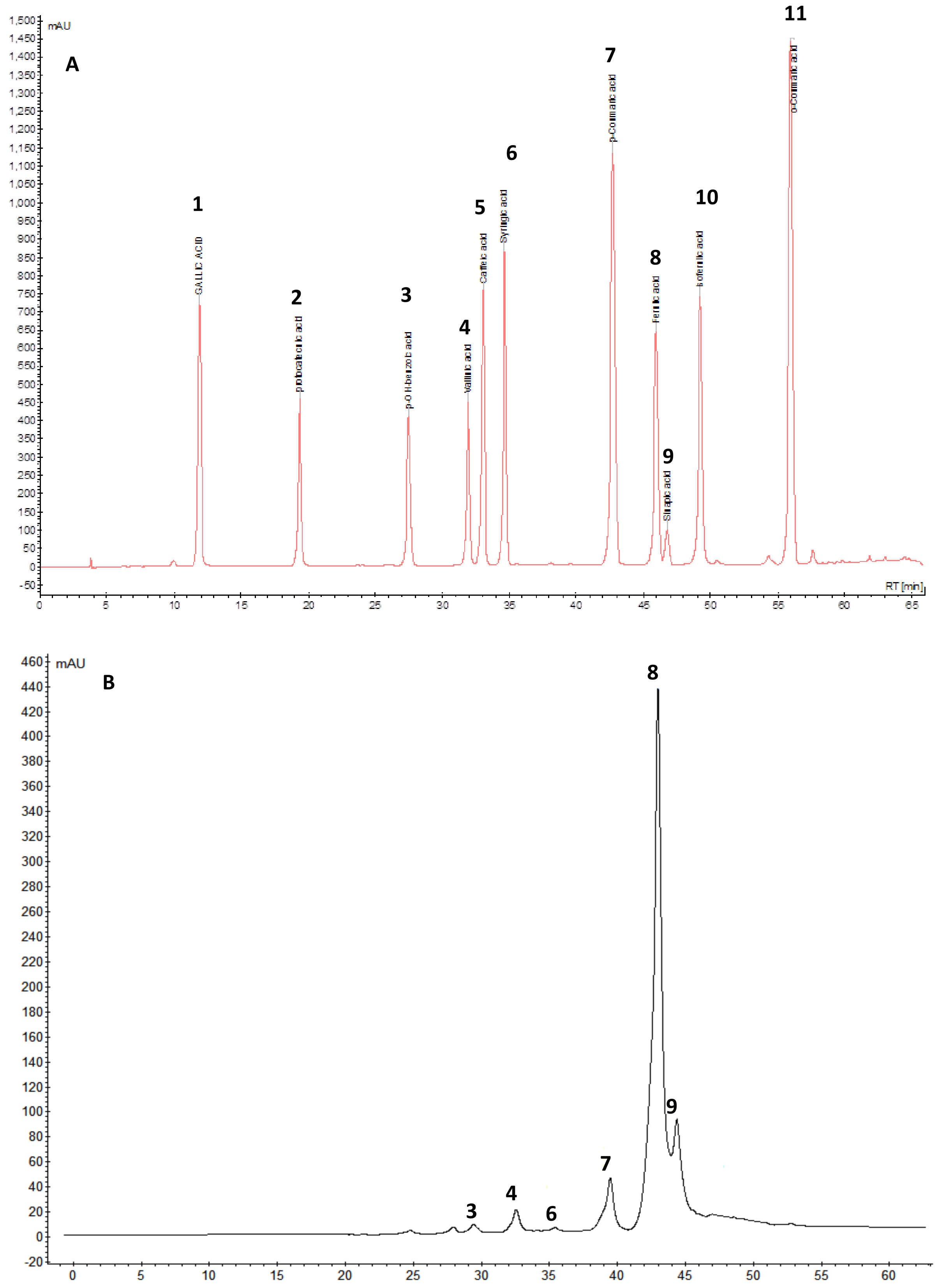
A
mAU
RT [min]
GALLIC ACID
Caffeic acid
Syringic acid
p-Coumaric acid
Ferulic acid
Sinapic acid
o-Coumaric acid
B
mAU

Supplemental figure 2: HPLC-UV/VIS chromatograms of phenolic acids.

A: Chromatogram of phenolic acids standards: 1 gallic acid; 2 protocatechuic acid; 3 *p*-hydroxybenzoic acid; 4 vanillic acid; 5 caffeic acid; 6 syringic acid; 7 *p*-coumaric acid; 8 ferulic acid; 9 sinapic acid; 10 isoferulic acid; 11 *o*-coumaric acid

B: Chromatogram of the bound fraction of Kennedy grown at 20°C, 3 *p*-hydroxybenzoic acid; 4 vanillic acid; 6 syringic acid; 7 *p*-coumaric acid; 8 ferulic acid; 9 sinapic acid.
